# Quiescent behavior in response to bacterial infection in *C. elegans*

**DOI:** 10.17912/micropub.biology.001412

**Published:** 2025-03-05

**Authors:** Daniel Moses, Carly A Needham, Hilary DeBardeleben

**Affiliations:** 1 Department of Biological Sciences, Duquesne University, Pittsburgh, Pennsylvania, United States; 2 Biology and Health Sciences, Commonwealth University - Bloomsburg

## Abstract

Sickness behaviors serve an important role in recovery from infection. Using the WorMotel and a motivated displacement assay, we show that *C. elegans* engages in quiescent behavior following infection with *Serratia marcesens* , a bacterial pathogen. This quiescence is increased with increasing severity of the infection. Furthermore, we show this behavior is distinct from stress-induced sleep due to a lack of feeding quiescence and regulation by sleep-inducing neurons.

**Figure 1. Locomotion quiescence in C. elegans following infection by virulent S. marcesens:**
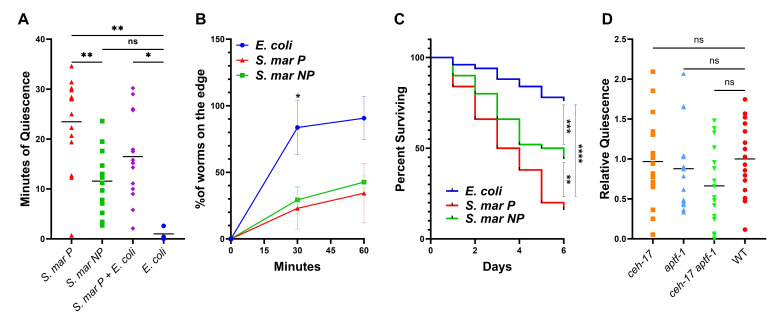
A. Minutes of quiescence in the first hour following 48 hours of exposure to *S. marcesens* pigmented ( *S. mar* P) (n=12), *S. marcescens* non-pigmented ( *S. mar* NP) (n=12), mixed *S. marcesens* pigmented and *E. coli* (n=12), or *E. coli* (n=4) *p<.05, **p<.01, based on Dunn's multiple comparisons test. B. Percentage of total worms removed from the edge of the plate at 30 and 60 minutes in a motivated displacement assay after 48 hours of exposure on *S. marcesens* pigmented (n=95), *S. marcescens* non-pigmented (n=126), or *E. coli* (n=102) (data points show mean of three trials with standard deviation) *p<.05 based on Tukey's multiple comparisons test. C. Survival assay showing living worms counted each day on each strain of *S. marcesens* and *E. coli* for six days (n=50 for each group) **p<.01, ***p<.001, ****p<.0001, based on log-rank (Mantel-Cox) test. D. Relative quiescence of mutant strains as compared to wild-type in the first hour following 48 hours of exposure to *S. marcesens* pigmented. All mutants are normalized to wild-type controls measured on the same WorMotel. No significant difference between wild-type and mutant *C. elegans* based on Dunn's multiple comparisons test (n=16 per group).

## Description

Sickness behaviors during bacterial infections are common in all animals and can increase survival (Hart 1988) . Here we have used *C. elegans* to investigate behaviors during infection and recovery from the pathogen *Serratia marcesens* . *S. marcesens* is an opportunistic pathogen in many animals. It infects the intestines of *C. elegans* leading to distension of the intestine and reduced survival compared to *E. coli* (Kurz et al., 2003) . Previous research has demonstrated that infection with another bacterial species, *Pseudomonas aeruginosa* , leads to stress-induced sleep in *C. elegans* (Pradhan et al., 2024) . In this study, we aimed to determine whether a similar response occurs following infection with *S. marcescens* .

Sleep is determined in *C. elegans* using several behavioral assays including locomotion quiescence, pumping quiescence, and reversibility (Hill et al 2014) . We measured locomotion quiescence as the first step in investigating stress-induced sleep. By removing *C. elegans* from the pathogen and recording behavior after infection using the motivated displacement assay (Chávez-Pérez et al., 2021) and the WorMotel (Churgin et al., 2019) , we were able to quantify locomotion quiescence similar to the stress-induced locomotion quiescence seen following UV irradiation (DeBardeleben et al., 2017) or heat shock (Hill et al., 2014) . The WorMotel is a microchip that separates individual worms for imaging. Pictures were taken every fifteen seconds. Image subtraction analysis determines activity levels for each worm based on pixel changes, with bouts of inactivity deemed quiescence (Churgin et al., 2019) . We found that imaging the *C. elegans* on the WorMotel during infection with *S. marcesens* did not show reduced activity. This is similar to what is seen during heat shock where worms are active during the heat shock but quiescent in the hour following (Hill et al., 2014) . Instead, we exposed adult *C. elegans* to two strains of *S. marcesens* or an *E. coli* control for 48 hours. After exposure, we put them either onto the WorMotel with *E.coli* as a food source or onto the motivated displacement assay. *C. elegans* showed a significant increase in minutes of quiescence in the first hour following exposure to *S. marcesens* as compared to *E. coli* when measured on the WorMotel ( Figure 1A ). This was confirmed by results of the motivated displacement assay. In this assay, we placed *C. elegans* in the center of a 10cm petri dish with nematode growth media and the food source, *E. coli* , placed in a ring around the edge. We then counted the number of worms that reached the edge every thirty minutes. After sixty minutes the worms that had not reached the edge were each touched with a wire pick to determine if the worms would respond to a stimulus (and were therefore still alive) (n=23, 55 and 51 for the *E.coli* , *S. marcesens* NP and *S. marcesens* P groups respectively). Any worm that did not respond was deemed dead and removed from the analysis. Living worms were counted in the total and data was plotted as a percentage of the total that reached the edge. More *C. elegans* exposed to *E. coli* were able to reach the edge at thirty minutes than the worms exposed to the pigmented strain of *S. marcesens* ( Figure 1B ). *C. elegans* ' response to mechanical touch in this assay demonstrated reversibility of the quiescence. Previous work has shown that *C. elegans* will engage in satiety-quiescence when moved from a low-nutrient food source to a high-nutrient food source (You et al., 2008) . The response to *S. marcesens* was not due to satiety quiescence since worms grown on a mix of *E. coli* and *S. marcesens* (and therefore well fed but still infected) were still quiescent following exposure to the pathogen ( Figure 1A ). Furthermore, we were able to show that the severity of infection is correlated with the duration of quiescence following infection. We used two strains of *S. marcesens* , pigmented and non-pigmented. We wanted a low and high virulence strain of the same species and found that this pigmented vs non-pigmented strain worked well in that it led to significant mortality in *C. elegans* in a relatively short period of time but was not a danger to humans. This makes the model of infection safer to use. The virulence of the pigmented strain was higher as indicated by significantly lower rates of survival in the presence of the pigmented strain as compared to the non-pigmented strain and *E. coli* ( Figure 1C ). The pigmented strain of *S. marcescens* also led to significantly more minutes of quiescence than the non-pigmented strain ( Figure 1A ).

To further investigate if this behavior was stress-induced sleep we measured feeding quiescence in *C. elegans* . We followed the same infection protocol described above and then counted the number of pharyngeal pumps in one minute for each worm (n=15 per group) after moving the animal from the pathogen to *E. coli* . Pumping rates were measured every twenty minutes for the first hour on *E. coli* . The animals infected with two strains of *S. marcesens* did not have significantly different pumping rates at any time point compared to the *E. coli* control when using Tukey's multiple comparisons test. Notably, the reduced pumping seen during infection by *P. aeruginosa* is very slight (Pradhan et al., 2024) and we may just be unable to measure a small difference via eye.

To further test if this behavior was similar to stress-induced sleep, we looked at neuronal regulation of this behavior. Stress-induced sleep in *C. elegans* is regulated by the ALA and RIS neurons. Genetic mutants in the *ceh-17* and *aptf-1* genes will not develop these neurons and have reduced quiescence following stress (Chávez-Pérez et al., 2021). We used the WorMotel to measure quiescence of the mutant strains lacking ALA (IB16: *ceh-17 ( np1 ) I)* and RIS (HBR232: *aptf-1 ( tm3287 ) II)* and a double mutant (NQ1065: *ceh-17 ( np1 ) I; aptf-1 ( tm3287 ) II)* that lacks both neurons following infection by the pigmented *S. marcesens* strain. We did not see significantly reduced quiescence in any of these mutants ( Figure 1D ).

The lack of pumping quiescence and regulation by ALA and RIS indicate that the locomotion quiescence we are seeing at this stage of infection is distinct from stress-induced sleep. Further work is needed to determine how this behavior is regulated and if it serves to improve survival in *C. elegans* following bacterial infection.

## Methods

*Bacterial Growth and Maintenance*

Both the *S. marcescens* and *E. coli* strains used were grown overnight in a 35°C incubator and maintained on 150mm x 50mm petri dishes containing tryptic soy agar. Broth cultures for spreading on petri dishes were also grown in tryptic soy broth overnight in a 37°C shaking incubator. The reagents table shows the bacterial strains used for this project.

*Infection with S. marcescens*

All bacteria were taken from fresh tryptic soy agar plates grown the day before, and grown overnight in tryptic soy broth as previously described. The following day 450μL of the broth was spread evenly throughout modified NGM petri dishes (150mm x 15mm) with 40% additional peptone (as used in (Tan et al., 1999) ). The plates were then incubated for 24 hrs. at 37°C and then left out at room temperature for 24hrs. Young adult *C. elegans* ( N2 ) were then placed onto the plates to allow for infection for 48 hours. Individual worms were picked from their respective plates directly onto the WorMotel for immediate imaging for one hour.

*Motivated Displacement Assay*

Petri dishes used for this assay were created by placing a thin line (~5mm) of *E. coli* HB101 around the edge of a 150mm x 15mm petri dish containing normal NGM. Adult *C. elegans* previously either infected with *S. marcescens* or kept on *E. coli* HB101 were washed into 15mL conical tubes with M9, and then washed an additional three times to remove as much bacteria as possible. Afterwards, a single drop of the M9 broth containing the *C. elegans* was placed onto a bacteria free NGM plate, which they were then picked off of and placed in the center of the petri dishes with *E. coli* HB101 around the edge. This ensured that the *C. elegans* were not inhibited by being stuck in the drop. Individuals that completely entered the edge with *E. coli* HB101 were removed and counted every 30 mins in the order in which they were placed. After 60 minutes, the number of individuals that had not reached the *E. coli* HB101 were counted.

*C. elegans Survival Assays*

Young adult *C. elegans* ( N2 ) were picked off plates with a sterile wire, and either introduced to a lawn of *S. marcescens* as previously described or their normal food source of *E. coli* HB101 grown in the same conditions. All of these plates contained the additional peptone as previously described. For all groups, adults were picked onto fresh plates daily to avoid contamination by subsequent generations. Any larval *C. elegans* were not picked onto new petri dishes and were not counted towards the number of survivors. Living *C. elegans* were defined as any that responded to a gentle tapping with a platinum wire or waving an eyelash with 190 proof ethanol in front of their pharynx.

*Pumping Assay*

Following the infection protocol, *C. elegans* were moved from the pathogen condition to E. coli lawns on NGM plates. Twenty, forty and sixty minutes after being placed on the lawn, pharyngeal pumps were counted for thirty seconds using 40x magnification on a brightfield microscope.

## Reagents

**Table:** 

**Species**	**Strain**	**Genotype**	**Source**	**Reference**
*E. coli*	DA837	Streptomycin-resistant OP50	CGC	(Davis et al. 1995)
*E. coli*	HB101	*[supE44 hsdS20(rB-mB-) recA13 ara-14 proA2 lacY1 galK2 rpsL20 xyl-5 mtl-1]*	CGC	(Davis et al. 1995)
*S. marcesens*	Pigmented		Drexel University	
*S. marcesens*	Non-Pigmented		ATCC	
*C. elegans*	N2	wild-type	CGC	(Brenner 1974)
*C. elegans*	IB16	*ceh-17 ( np1 ) I *	CGC	(Hill et al. 2014)
*C. elegans*	HBR232	*aptf-1 ( tm3287 ) II *	CGC	(Turek et al. 2013)
*C. elegans*	NQ1065	*ceh-17 ( np1 ) I; aptf-1 ( tm3287 ) II *	Raizen Lab	(Grubbs et al. 2020)
